# 2-*N*-Benzyl-2,6-dide­oxy-2,6-imino-3,4-*O*-isopropyl­idene-3-*C*-methyl-d-allono­nitrile

**DOI:** 10.1107/S1600536812016273

**Published:** 2012-04-21

**Authors:** Benjamin J. Ayers, Sarah F. Jenkinson, George W. J. Fleet, Amber L. Thompson

**Affiliations:** a Department of Organic Chemistry, Chemistry Research Laboratory, University of Oxford, Oxford OX1 3TA, England; b Department of Chemical Crystallography, Chemistry Research Laboratory, University of Oxford, Oxford OX1 3TA, England

## Abstract

X-ray crystallography firmly established the relative stereochemistry of the title compound, C_17_H_22_N_2_O_3_. The absolute configuration was determined by use of 2-*C*-methyl-d-ribonolactone as the starting material. The compound exists as O—H⋯N hydrogen-bonded chains of mol­ecules running parallel to the *a*-axis.

## Related literature

For 2-*C*-methyl sugar lactones and their use in synthesis, see: da Cruz *et al.* (2011 ▶); Best *et al.* (2010 ▶); da Cruz & Horne (2008 ▶6); Booth *et al.* (2008 ▶); Hotchkiss, Soengas *et al.* (2007 ▶); Hotchkiss, Kato *et al.* (2007 ▶); Hotchkiss *et al.* (2006 ▶); Sowden & Strobach (1960 ▶). For the biological activity of polyhy­droxy­lated piperidines, see: Nash *et al.* (2011 ▶); Watson *et al.* (2001 ▶). For the extinction correction, see: Larson (1970 ▶). For the temperature controller, see: Cosier & Glazer (1986 ▶).
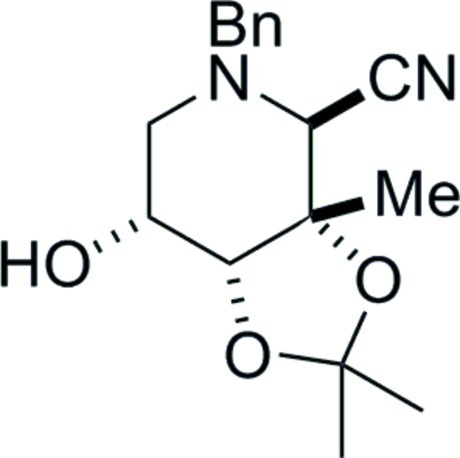


## Experimental

### 

#### Crystal data

C_17_H_22_N_2_O_3_
*M* *_r_* = 302.37
Orthorhombic, 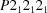

*a* = 8.5647 (3) Å
*b* = 10.0019 (4) Å
*c* = 18.7031 (7) Å
*V* = 1602.17 (10) Å^3^
*Z* = 4
Mo *K*α radiation
μ = 0.09 mm^−1^
*T* = 150 K
0.25 × 0.25 × 0.20 mm

#### Data collection

Nonius KappaCCD diffractometer
Absorption correction: multi-scan (*DENZO*/*SCALEPACK*; Otwinowski & Minor, 1997 ▶) *T*_min_ = 0.96, *T*_max_ = 0.98
7953 measured reflections
2082 independent reflections
1808 reflections with *I* > 2σ(*I*)
*R*_int_ = 0.042

#### Refinement

*R*[*F* ^2^ > 2σ(*F* ^2^)] = 0.041
*wR*(*F* ^2^) = 0.106
*S* = 0.97
2082 reflections
200 parameters
H-atom parameters constrained
Δρ_max_ = 0.22 e Å^−3^
Δρ_min_ = −0.22 e Å^−3^

### 

Data collection: *COLLECT* (Nonius, 2001 ▶).; cell refinement: *DENZO*/*SCALEPACK* (Otwinowski & Minor, 1997 ▶); data reduction: *DENZO*/*SCALEPACK*; program(s) used to solve structure: *SIR92* (Altomare *et al.*, 1994 ▶); program(s) used to refine structure: *CRYSTALS* (Betteridge *et al.*, 2003 ▶); molecular graphics: *CAMERON* (Watkin *et al.*, 1996 ▶); software used to prepare material for publication: *CRYSTALS*.

## Supplementary Material

Crystal structure: contains datablock(s) global, I. DOI: 10.1107/S1600536812016273/lh5456sup1.cif

Structure factors: contains datablock(s) I. DOI: 10.1107/S1600536812016273/lh5456Isup2.hkl

Additional supplementary materials:  crystallographic information; 3D view; checkCIF report

## Figures and Tables

**Table 1:** Hydrogen-bond geometry (Å, °)

*D*—H⋯*A*	*D*—H	H⋯*A*	*D*⋯*A*	*D*—H⋯*A*
O1—H11⋯N4^i^	0.88	2.15	2.997 (3)	161

Symmetry code: (i) 
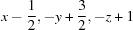
.

**Fig. 1.**
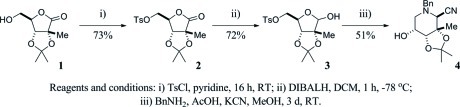
Synthetic Scheme

**Fig. 2.**
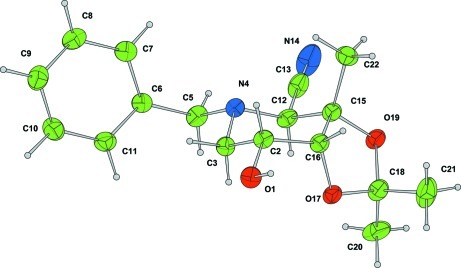
The title compound with displacement ellipsoids drawn at the 50% probability level. H atoms are shown as spheres of arbitary radius.

**Fig. 3.**
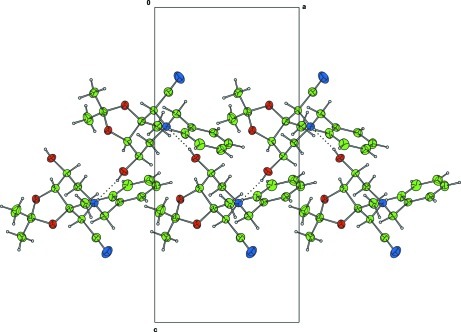
Packing diagram for the crystal projected along the b-axis. Hydrogen bonds are shown as dotted lines.

## supplementary crystallographic information

### Comment

Many polyhydroxylated piperidines have been found to display interesting biological properties (Nash *et al.*, 2011; Watson *et al.*, 2001). 2-*C*-Methyl lactones, derived from sugars (Hotchkiss, Soengas *et al.*, 2007; Sowden & Strobach, 1960; Hotchkiss *et al.*, 2006), have been used for the synthesis of iminosugars bearing a carbon branch (da Cruz *et al.*, 2011; Best *et al.*, 2010; da Cruz *et al.*, 2008; Hotchkiss, Kato *et al.*, 2007). In a new one-pot approach to carbon-branched piperidines, the α-iminonitrile 4 was prepared from the lactol tosylate 3, itself readily available in two steps from 2-*C*-methyl-D-ribonolactone 1 (Booth *et al.*, 2008), by Strecker α-aminonitrile formation and concomitant intramolecular tosylate displacement (Fig. 1).

X-ray crystallography firmly established the relative stereochemistry of the title compound 4. The absolute configuration was determined by the use of 2-*C*-methyl-D-ribonolactone 1 as the starting material. The acetonide ring adopts an envelope conformation with C16 out of the plane and the piperidine ring adopts a chair conformation (Fig. 2). The compound exists as O—H···N hydrogen-bonded chains of molecules running parallel to the *a*-axis (Fig. 3). Only classical hydrogen-bonding was considered.

### Experimental

α-Iminonitrile 4 was recrystallized by diffusion from a mixture of ethyl acetate and cyclohexane: m.p. 394–395 K; [α]_D_^20^ +39.7 (*c* 5.5, methanol).

### Refinement

In the absence of significant anomalous scattering, Friedel pairs were merged and the absolute configuration was assigned from the starting material.

The H atoms were all located in a difference map, but those attached to carbon atoms were repositioned geometrically. The H atoms were initially refined with soft restraints on the bond lengths and angles to regularize their geometry (C—H in the range 0.93–0.98, O—H = 0.82 Å) and *U*_iso_(H) (in the range 1.2–1.5 times *U*_eq_ of the parent atom), after which the positions were refined with riding constraints.

### Crystal data

**Table:**

C_17_H_22_N_2_O_3_	*F*(000) = 648
*M**_r_* = 302.37	*D*_x_ = 1.253 Mg m^−^^3^
Orthorhombic, *P*2_1_2_1_2_1_	Mo *K*α radiation, λ = 0.71073 Å
Hall symbol: P 2ac 2ab	Cell parameters from 1959 reflections
*a* = 8.5647 (3) Å	θ = 5–27°
*b* = 10.0019 (4) Å	µ = 0.09 mm^−^^1^
*c* = 18.7031 (7) Å	*T* = 150 K
*V* = 1602.17 (10) Å^3^	Block, colourless
*Z* = 4	0.25 × 0.25 × 0.20 mm

### Data collection

**Table:**

Nonius KappaCCD diffractometer	1808 reflections with *I* > 2σ(*I*)
Graphite monochromator	*R*_int_ = 0.042
ω scans	θ_max_ = 27.5°, θ_min_ = 5.2°
Absorption correction: multi-scan (*DENZO*/*SCALEPACK*; Otwinowski & Minor, 1997)	*h* = −11→11
*T*_min_ = 0.96, *T*_max_ = 0.98	*k* = −12→12
7953 measured reflections	*l* = −24→24
2082 independent reflections	

### Refinement

**Table:**

Refinement on *F*^2^	Hydrogen site location: inferred from neighbouring sites
Least-squares matrix: full	H-atom parameters constrained
*R*[*F*^2^ > 2σ(*F*^2^)] = 0.041	Method = Modified Sheldrick *w* = 1/[σ^2^(*F*^2^) + (0.07*P*)^2^ + 0.23*P*], where *P* = (max(*F*_o_^2^,0) + 2*F*_c_^2^)/3
*wR*(*F*^2^) = 0.106	(Δ/σ)_max_ = 0.0002994
*S* = 0.97	Δρ_max_ = 0.22 e Å^−^^3^
2082 reflections	Δρ_min_ = −0.22 e Å^−^^3^
200 parameters	Extinction correction: Larson (1970), Equation 22
0 restraints	Extinction coefficient: 280 (110)
Primary atom site location: structure-invariant direct methods	

### Special details

**Table:**

Experimental. The crystal was placed in the cold stream of an Oxford Cryosystems open-flow nitrogen cryostat (Cosier & Glazer, 1986) with a nominal stability of 0.1 K.

### Fractional atomic coordinates and isotropic or equivalent isotropic displacement parameters (Å^2^)

**Table:**

	*x*	*y*	*z*	*U*_iso_*/*U*_eq_	
O1	0.28415 (17)	0.66972 (14)	0.47877 (7)	0.0285	
C2	0.3904 (2)	0.7319 (2)	0.52685 (10)	0.0240	
C3	0.4656 (2)	0.62006 (19)	0.56970 (10)	0.0245	
N4	0.58010 (19)	0.67473 (16)	0.62140 (8)	0.0237	
C5	0.6509 (2)	0.5619 (2)	0.66204 (10)	0.0290	
C6	0.7562 (2)	0.47284 (19)	0.61857 (10)	0.0252	
C7	0.9075 (2)	0.5134 (2)	0.60165 (11)	0.0298	
C8	1.0092 (3)	0.4276 (2)	0.56690 (11)	0.0356	
C9	0.9612 (3)	0.2999 (2)	0.54895 (12)	0.0377	
C10	0.8116 (3)	0.2586 (2)	0.56503 (13)	0.0377	
C11	0.7094 (3)	0.3444 (2)	0.59975 (12)	0.0317	
C12	0.4923 (2)	0.7579 (2)	0.67274 (10)	0.0258	
C13	0.5940 (3)	0.8056 (2)	0.73119 (12)	0.0349	
N14	0.6690 (3)	0.8455 (3)	0.77702 (12)	0.0563	
C15	0.4085 (2)	0.8802 (2)	0.63836 (10)	0.0258	
C16	0.3102 (2)	0.8340 (2)	0.57458 (10)	0.0251	
O17	0.17610 (16)	0.77936 (14)	0.60920 (7)	0.0267	
C18	0.1432 (2)	0.8661 (2)	0.66828 (11)	0.0322	
O19	0.29237 (16)	0.92224 (14)	0.68874 (8)	0.0293	
C20	0.0777 (3)	0.7836 (3)	0.72865 (12)	0.0435	
C21	0.0377 (3)	0.9798 (3)	0.64619 (15)	0.0503	
C22	0.5170 (3)	0.9967 (2)	0.62255 (12)	0.0337	
H21	0.4744	0.7726	0.4999	0.0256*	
H31	0.5218	0.5592	0.5364	0.0293*	
H32	0.3838	0.5684	0.5958	0.0277*	
H51	0.5641	0.5120	0.6821	0.0339*	
H52	0.7138	0.6041	0.7009	0.0357*	
H71	0.9426	0.5985	0.6148	0.0359*	
H81	1.1119	0.4591	0.5562	0.0416*	
H91	1.0304	0.2415	0.5259	0.0458*	
H101	0.7821	0.1690	0.5530	0.0463*	
H111	0.6056	0.3166	0.6118	0.0379*	
H121	0.4100	0.7095	0.6953	0.0311*	
H161	0.2829	0.9145	0.5454	0.0290*	
H203	0.0584	0.8381	0.7698	0.0637*	
H202	−0.0209	0.7457	0.7123	0.0635*	
H201	0.1521	0.7143	0.7417	0.0639*	
H211	0.0220	1.0417	0.6876	0.0718*	
H212	−0.0621	0.9372	0.6318	0.0712*	
H213	0.0838	1.0319	0.6067	0.0702*	
H223	0.4563	1.0631	0.5969	0.0499*	
H222	0.6019	0.9635	0.5928	0.0502*	
H221	0.5578	1.0406	0.6665	0.0511*	
H11	0.2417	0.7301	0.4504	0.0457*	

### Atomic displacement parameters (Å^2^)

**Table:**

	*U*^11^	*U*^22^	*U*^33^	*U*^12^	*U*^13^	*U*^23^
O1	0.0278 (7)	0.0332 (7)	0.0244 (6)	0.0012 (7)	−0.0070 (6)	−0.0039 (6)
C2	0.0225 (10)	0.0293 (10)	0.0201 (8)	−0.0011 (8)	−0.0021 (8)	−0.0007 (8)
C3	0.0242 (9)	0.0254 (9)	0.0239 (9)	0.0009 (8)	−0.0016 (8)	0.0003 (8)
N4	0.0231 (8)	0.0267 (8)	0.0215 (7)	0.0005 (7)	−0.0002 (7)	−0.0002 (7)
C5	0.0277 (10)	0.0346 (11)	0.0249 (9)	0.0025 (9)	−0.0021 (8)	0.0032 (9)
C6	0.0253 (9)	0.0272 (10)	0.0233 (9)	0.0004 (8)	−0.0041 (8)	0.0050 (8)
C7	0.0295 (10)	0.0318 (10)	0.0281 (10)	−0.0015 (9)	−0.0036 (9)	0.0040 (9)
C8	0.0264 (11)	0.0433 (12)	0.0370 (11)	0.0009 (10)	0.0014 (9)	0.0064 (10)
C9	0.0368 (12)	0.0367 (12)	0.0397 (12)	0.0081 (10)	0.0054 (10)	0.0025 (10)
C10	0.0386 (12)	0.0288 (10)	0.0457 (12)	0.0000 (10)	−0.0020 (11)	−0.0007 (10)
C11	0.0254 (10)	0.0320 (10)	0.0377 (11)	−0.0020 (10)	−0.0031 (9)	0.0022 (9)
C12	0.0202 (9)	0.0347 (10)	0.0226 (8)	−0.0004 (9)	−0.0014 (8)	−0.0006 (8)
C13	0.0287 (10)	0.0480 (13)	0.0280 (10)	0.0089 (10)	−0.0025 (9)	−0.0087 (10)
N14	0.0397 (12)	0.0796 (16)	0.0496 (13)	0.0170 (13)	−0.0156 (10)	−0.0290 (13)
C15	0.0225 (9)	0.0295 (10)	0.0254 (9)	−0.0005 (9)	0.0015 (8)	−0.0028 (8)
C16	0.0233 (9)	0.0274 (9)	0.0246 (9)	0.0006 (8)	−0.0010 (7)	0.0000 (8)
O17	0.0214 (7)	0.0322 (7)	0.0266 (7)	−0.0012 (6)	0.0021 (6)	−0.0085 (6)
C18	0.0240 (10)	0.0411 (12)	0.0314 (11)	0.0006 (9)	−0.0023 (9)	−0.0138 (9)
O19	0.0219 (7)	0.0362 (8)	0.0297 (7)	−0.0004 (6)	0.0010 (6)	−0.0104 (6)
C20	0.0300 (12)	0.0660 (16)	0.0346 (11)	−0.0147 (12)	0.0082 (10)	−0.0152 (12)
C21	0.0372 (13)	0.0574 (16)	0.0563 (15)	0.0182 (13)	−0.0146 (12)	−0.0231 (13)
C22	0.0346 (11)	0.0305 (10)	0.0362 (11)	−0.0075 (10)	0.0036 (10)	−0.0040 (9)

### Geometric parameters (Å, º)

**Table:**

O1—C2	1.422 (2)	C11—H111	0.958
O1—H11	0.882	C12—C13	1.477 (3)
C2—C3	1.519 (3)	C12—C15	1.557 (3)
C2—C16	1.521 (3)	C12—H121	0.954
C2—H21	0.968	C13—N14	1.143 (3)
C3—N4	1.482 (2)	C15—C16	1.531 (3)
C3—H31	0.994	C15—O19	1.433 (2)
C3—H32	0.998	C15—C22	1.519 (3)
N4—C5	1.490 (2)	C16—O17	1.427 (2)
N4—C12	1.477 (2)	C16—H161	0.999
C5—C6	1.506 (3)	O17—C18	1.433 (2)
C5—H51	0.971	C18—O19	1.447 (2)
C5—H52	0.998	C18—C20	1.507 (3)
C6—C7	1.394 (3)	C18—C21	1.510 (3)
C6—C11	1.391 (3)	C20—H203	0.958
C7—C8	1.385 (3)	C20—H202	0.975
C7—H71	0.935	C20—H201	0.972
C8—C9	1.383 (3)	C21—H211	1.000
C8—H81	0.956	C21—H212	0.992
C9—C10	1.380 (4)	C21—H213	0.987
C9—H91	0.937	C22—H223	0.971
C10—C11	1.387 (3)	C22—H222	0.974
C10—H101	0.958	C22—H221	0.995
			
C2—O1—H11	110.2	N4—C12—H121	112.2
O1—C2—C3	106.45 (16)	C13—C12—H121	105.8
O1—C2—C16	112.06 (16)	C15—C12—H121	103.9
C3—C2—C16	112.12 (15)	C12—C13—N14	177.7 (2)
O1—C2—H21	109.3	C12—C15—C16	109.79 (16)
C3—C2—H21	105.6	C12—C15—O19	106.20 (15)
C16—C2—H21	111.0	C16—C15—O19	102.67 (15)
C2—C3—N4	110.68 (15)	C12—C15—C22	113.61 (16)
C2—C3—H31	109.0	C16—C15—C22	114.56 (16)
N4—C3—H31	108.3	O19—C15—C22	109.11 (16)
C2—C3—H32	110.0	C15—C16—C2	114.30 (16)
N4—C3—H32	109.6	C15—C16—O17	101.79 (14)
H31—C3—H32	109.2	C2—C16—O17	111.85 (16)
C3—N4—C5	108.84 (15)	C15—C16—H161	108.1
C3—N4—C12	107.17 (15)	C2—C16—H161	109.1
C5—N4—C12	107.60 (14)	O17—C16—H161	111.5
N4—C5—C6	114.61 (15)	C16—O17—C18	106.03 (14)
N4—C5—H51	105.9	O17—C18—O19	105.37 (15)
C6—C5—H51	111.3	O17—C18—C20	108.63 (18)
N4—C5—H52	105.7	O19—C18—C20	110.04 (17)
C6—C5—H52	108.7	O17—C18—C21	111.28 (18)
H51—C5—H52	110.5	O19—C18—C21	107.97 (18)
C5—C6—C7	120.47 (18)	C20—C18—C21	113.3 (2)
C5—C6—C11	120.68 (19)	C18—O19—C15	108.96 (14)
C7—C6—C11	118.6 (2)	C18—C20—H203	110.8
C6—C7—C8	120.7 (2)	C18—C20—H202	107.5
C6—C7—H71	120.2	H203—C20—H202	108.8
C8—C7—H71	119.0	C18—C20—H201	109.6
C7—C8—C9	120.0 (2)	H203—C20—H201	108.5
C7—C8—H81	118.2	H202—C20—H201	111.7
C9—C8—H81	121.8	C18—C21—H211	109.6
C8—C9—C10	120.0 (2)	C18—C21—H212	105.4
C8—C9—H91	120.0	H211—C21—H212	111.2
C10—C9—H91	120.1	C18—C21—H213	111.3
C9—C10—C11	120.2 (2)	H211—C21—H213	107.9
C9—C10—H101	118.3	H212—C21—H213	111.6
C11—C10—H101	121.5	C15—C22—H223	107.1
C6—C11—C10	120.5 (2)	C15—C22—H222	107.8
C6—C11—H111	118.4	H223—C22—H222	110.5
C10—C11—H111	121.1	C15—C22—H221	113.1
N4—C12—C13	111.28 (16)	H223—C22—H221	107.1
N4—C12—C15	114.13 (15)	H222—C22—H221	111.1
C13—C12—C15	108.91 (17)		

### Hydrogen-bond geometry (Å, º)

**Table:**

*D*—H···*A*	*D*—H	H···*A*	*D*···*A*	*D*—H···*A*
C22—H222···O1^i^	0.97	2.45	3.405 (3)	167
O1—H11···N4^ii^	0.88	2.15	2.997 (3)	161

Symmetry codes: (i) *x*+1/2, −*y*+3/2, −*z*+1; (ii) *x*−1/2, −*y*+3/2, −*z*+1.
